# A cluster of Mayaro virus infections in a film team returning from Suriname, February 2024

**DOI:** 10.2807/1560-7917.ES.2024.29.44.2400679

**Published:** 2024-10-31

**Authors:** Hans Martin Orth, Stefanie Pfau, Martin Gabriel, Stephan Günther, Dennis Tappe, Daniel Hornuss, Irmela Müller-Stöver, Martha Charlotte Holtfreter, Tom Luedde, Jonas Schmidt-Chanasit, Torsten Feldt

**Affiliations:** 1 Department of Gastroenterology, Hepatology and Infectious Diseases, Medical Faculty and University Hospital Düsseldorf, Heinrich-Heine-University Düsseldorf, Düsseldorf, Germany; 2 Department of Medicine II, Division of Infectious Diseases, Medical Centre-University Hospital Freiburg, Faculty of Medicine, Freiburg, Germany; 3 Bernhard Nocht Institute for Tropical Medicine, Hamburg, Germany; 4 University of Hamburg, Faculty of Mathematics, Informatics and Natural Sciences, Hamburg, Germany

**Keywords:** Alphavirus, Mayaro, Suriname, Germany, vector-borne infections, viral infections, imported viral diseases, travel

## Abstract

Mayaro virus is endemic to the tropical Americas, where the incidence is currently increasing. Like other viruses of the Semliki Forest virus serocomplex, such as *Alphavirus chikungunya*, symptomatic infections are typically characterised by an acute febrile disease followed by long-lasting arthralgia. Cases in travellers are rarely reported but may be underdiagnosed. We report on four people who diagnosed with Mayaro fever after working in remote areas of Suriname as members of a film team.

The arthropod-borne *Alphavirus mayaro* or Mayaro virus (MAYV) is endemic in Central and South America and the Caribbean, but the majority of human infections occur in the Amazon rain forest. Humans most often acquire the infection through bites of the canopy-dwelling mosquito *Haemagogus janthinomys*, with nonhuman primates and other vertebrates playing a major role as animal reservoir [1]. While Brazil, the country reporting most cases worldwide, has just recently reported an increase of cases, particularly in the federal states of Amazonas and Pará, the latter bordering Suriname [2], travel-associated cases have only rarely been reported.

We report on a travel-associated cluster of MAYV infection in four members of a film crew during a stay in Suriname in February 2024, presenting after their return to two outpatient departments specialised in tropical medicine or infectious diseases.

## Outbreak setting

The film crew consisted of ca 40 people, and filming took place from 6 February 2024 at various locations along the Suriname River. Accommodations were basic, with some individuals sleeping in hammocks with bed nets outside of tents or buildings. The four patients described in this report had travelled to Suriname at different times. All four, however, arrived at the first filming spot on 6 February, where they had many mosquito bites despite the use of repellents. In all four, initial symptoms appeared around 7 days later.

## Patient 1

On 13 February, 7 days after her arrival in Suriname, a female in her thirties complained of a 3-day episode of fever accompanied by painful swelling of finger and foot joints and ankles. According to the patient, dengue and malaria were ruled out at a local health centre but no documentation is available. Symptomatic treatment with paracetamol was initiated. After a slight improvement, the pain increased again over the next days. Upon return to Germany, she contacted a tropical medicine outpatient department 10 days after symptom onset. At that time, most symptoms had subsided. However, she continued to experience notable joint pain, particularly in her hands and feet during the morning, which caused movement restrictions and only partially responded to non-steroidal anti-inflammatory drugs (NSAID). She reported that she intermittently experienced pain-free days. Laboratory test results were unremarkable apart from mild elevation of alanine aminotransferase (ALT) and aspartate aminotransferase (AST). Serology was negative for anti-chikungunya virus (CHIKV) IgM and IgG. Due to the local endemicity and the recent increase of cases, we additionally tested for MAYV, which revealed high IgM and IgG antibody titres (Table).

**Table:** Clinical and serological characteristics of patients with Mayaro virus returning from Suriname, 2024 (n = 4)

Characteristics	Patient 1	Patient 2	Patient 3	Patient 4
Symptom onset	13 Feb	Mid-Feb	Mid-Feb	14 Feb
Arthralgia				
Fingers, hands	Yes	Yes	Yes	Yes
Knees	Yes	Yes	No	Yes
Ankles	Yes	Yes	Yes	Yes
Feet	Yes	No	Yes	Yes
Serological tests				
Days post symptom onset	10	26^a^	61^a^	9
Anti-DENV IgM	Neg	Neg	Neg	NA
Anti-DENV IgG	**1:160**	**1:1,280**	Neg	NA
Anti-ZIKV IgM	Neg	Neg	Neg	NA
Anti-ZIKV IgG	**1:40**	**1:1,280**	Neg	NA
Anti-*Leptospira* IgM	NA	**Pos**	**Pos**	NA
Anti-*Leptospira* IgG	NA	Neg	Neg	NA
Anti-MAYV IgM	**1:640**	**1:320**	**1:640**	NA
Anti-MAYV IgG	**1:640**	**1:10,240**	**1:2,560**	NA
Anti-CHIKV IgM	Neg	Neg	Neg	**Pos**
Anti-CHIKV IgG	Neg	**1:80**	**1:640**	Neg
Days post symptom onset	23	145^a^	79^a^	103
Anti-MAYV IgM	**1:320**	Neg	**1:20**	Neg
Anti-MAYV IgG	**1:10,240**	**1:20,480**	**> 1:20,480**	**1:20,480**
Anti-CHIKV IgM	Neg	Neg	Neg	Neg
Anti-CHIKV IgG	**1:160**	**1:320**	**1:2,560**	**1:1,280**

CHIKV: chikungunya virus; DENV: dengue virus; MAYV: Mayaro virus; NA: not analysed; Neg: negative; Pos: positive; ZIKV: Zika virus.

^a^ An approximation of the number of days post symptom onset.

Test results considered positive are marked in bold. For CHIKV, DENV, MAYV and ZIKV, a result ≥ 1:20 was considered positive.

## Patient 2

Around mid-February, about 7 days after her arrival in Suriname, a female in her twenties complained of fever, malaise, diarrhoea, generalised rash and severe arthralgia in hands, feet, ankles and knees. Gastrointestinal symptoms and fever subsided after few days. The joint pain was partly immobilising. Four weeks after the symptom onset, Patient 2 visited the same tropical medicine outpatient department as Patient 1. At this time, laboratory test results were unremarkable, except for mild elevation of C-reactive protein (CRP). Serology was positive for anti-CHIKV IgG and anti-MAYV IgM and IgG (Table). In her medical history, she had been diagnosed with juvenile rheumatoid arthritis, but did not currently receive treatment. Severe joint pains persisted until mid-June. By mid-July, she still reported persisting fatigue leading to a need of sleep of up to 16 hours per day without major recovery.

## Patient 3

A female in her thirties experienced malaise, diarrhoea and abdominal pain around 14 days after her arrival in Suriname. The following day, fever, joint pain and swelling of the fingers, wrists and ankles appeared, followed by an erythema on the palms and on the soles of her feet, which progressed to a non-itching exanthema of the whole body except the head. At a local healthcare centre, symptomatic treatment with intravenous (i.v.) fluid and glucocorticoids were administered. Four days after return to Germany and 10 days after symptom onset, Patient 3 presented to the infectious disease outpatient department of a university clinic in south-west Germany. At that time, she still experienced considerable joint pains and fluctuating swelling of the fingers and wrists as well as fatigue. Laboratory investigations showed a mild elevation of lactate dehydrogenase (LDH), ALT and AST. The serology was positive for anti-CHIKV IgG, anti-MAYV IgM and IgG (Table). The joint pain and swellings persisted until June 2024.

## Patient 4

A female in her forties reported symptom onset on the eighth day of her stay in Suriname with malaise, nausea and vomiting. Incipient dehydration was treated at a local healthcare centre with i.v. fluids. Diarrhoea, fever and joint pain developed the following day followed by a generalised rash and dyspnoea a few days later. Upon return to her place of residence in Asia, a serological test conducted in her hometown was positive for anti-CHIKV IgM. Serology for MAYV was conducted 3 months later at the same specialised tropical medicine clinic as for Patients 1 and 2, with elevated anti-MAYV IgG and negative anti-MAYV IgM (Table). Most symptoms subsided after ca 10 days; only arthralgia of the feet, hands and ankles lasted for about 4 months and then gradually decreased.

According to information from the four patients, at least one more person of their team complained of similar symptoms, however, we could not obtain further information. Another patient treated for different symptoms tested negative for MAYV.

## Discussion

Chikungunya virus is the most frequently diagnosed virus of the Semliki Forest serocomplex in the Americas since the first description of autochthonous transmission in 2013 [3,4]. As these viruses show a strong serological cross-reactivity and cause a similar clinical picture, MAYV infection may often be misdiagnosed as CHIKV infection, while in patients with negative CHIKV serology (as in one of the four patients), Mayaro fever is only rarely considered as a differential diagnosis. The viraemic phase is usually no longer than 3–5 days [5], and in two patients where attempted, PCR for both CHIKV and MAYV returned negative. Serological follow-up with proof of different antibody kinetics has therefore been suggested to confirm the diagnosis [3,5]. We could show positive anti-MAYV IgM and negative anti-CHIKV IgM in the three patients who presented early after symptom onset, followed by stable high anti-MAYV IgG and only mild increase of anti-CHIKV IgG during follow-up in all four patients. We interpret the low IgG titres for flaviviruses including DENV and ZIKV as cross-reactivity to a previous vaccination against yellow fever, which all four patients had received before travel. Interestingly, IgM against *Leptospira* species were positive in two patients tested, with IgG remaining negative on follow-up. We assumed unspecific positivity due to a generalised immune activation.

Symptom onset was about 7 days after arrival at the first filming location on the banks of the Suriname River, ca 50 km upstream from the capital Paramaribo, where all four patients reported a high number of mosquito bites. It can therefore be assumed, that infection took place there. The estimated incubation period of ca 7 days goes in line with data from the literature ranging from few days up to 2 weeks [6].

Consistent with the literature, all four patients presented with an initial febrile episode followed by prolonged severe and partially immobilising arthralgia affecting the hands, ankles and feet. Other symptoms, such as rash, were not present in all patients. Particularly the gastrointestinal symptoms, which three of the four patients experienced, may not be clearly attributed to MAYV infection, but may also be due to concomitant gastrointestinal infections.

As by mid-July, one patient reported ongoing, undulating pain in the affected joints. The pain was particularly severe in the morning and only partly responsive to NSAID. Antirheumatic treatment (e.g. corticosteroids, methotrexate) may be considered depending on the further course, analogous to treatment of CHIKV infection [7].

Until now, only a few cases of MAYV infection have been reported in European travellers. A databank survey revealed 10 imported cases [6,8-14]. Apart from infections in a Dutch couple, most publications describe single sporadic cases [8]. Our case series therefore emphasises the potentially increasing importance of Mayaro fever as differential diagnosis in returning travellers with fever, rash and persistent joint pain. The limited knowledge of Mayaro fever outside the endemic areas, as well as the diagnostic challenges, may lead to considerable underdiagnosis of the disease.

At present, MAYV is only endemic to South and Central America including the Caribbean, likely due to its close association with the primary vector, *Haemagogus janthinomys*. The possibility of an urban infection cycle, as observed in yellow fever, however, is under discussion [1]. *Anopheles quadrimaculatus*, *Aedes aegypti* and *Aedes albopictus* were shown to be competent vectors under laboratory conditions [15-17]. In *A. aegypti*, vertical transmission of MAYV was shown under natural conditions [18] which highlights the potential of MAYV for spreading to regions where these vectors are present.

## Conclusion

Physicians should be aware of Mayaro fever and test travellers who present with symptoms of acute febrile disease followed by long-lasting arthralgia after returning from endemic regions. As no vaccination against MAYV is available, prophylactic measures are limited to protection from mosquito bites.
